# Modified Liu estimators in the linear regression model: An application to Tobacco data

**DOI:** 10.1371/journal.pone.0259991

**Published:** 2021-11-22

**Authors:** Iqra Babar, Hamdi Ayed, Sohail Chand, Muhammad Suhail, Yousaf Ali Khan, Riadh Marzouki

**Affiliations:** 1 College of Statistical and Actuarial Sciences, University of the Punjab, Lahore, Pakistan; 2 Department of Civil Engineering, College of Engineering, King Khalid University, Abha, KSA; 3 Department of Statistics, The University of Agriculture Peshawar, Amir Muhammad Khan Campus, Mardan, Pakistan; 4 Department of Mathematics and Statistics, Hazara University Mansehra, Mansehra, Pakistan; 5 Department of Chemistry, College of Science, King Khalid University, Abha, KSA; Vellore Institute of Technology: VIT University, INDIA

## Abstract

**Background:**

The problem of multicollinearity in multiple linear regression models arises when the predictor variables are correlated among each other. The variance of the ordinary least squared estimator become unstable in such situation. In order to mitigate the problem of multicollinearity, Liu regression is widely used as a biased method of estimation with shrinkage parameter ‘*d*’. The optimal value of shrinkage parameter plays a vital role in bias-variance trade-off.

**Limitation:**

Several estimators are available in literature for the estimation of shrinkage parameter. But the existing estimators do not perform well in terms of smaller mean squared error when the problem of multicollinearity is high or severe.

**Methodology:**

In this paper, some new estimators for the shrinkage parameter are proposed. The proposed estimators are the class of estimators that are based on quantile of the regression coefficients. The performance of the new estimators is compared with the existing estimators through Monte Carlo simulation. Mean squared error and mean absolute error is considered as evaluation criteria of the estimators. Tobacco dataset is used as an application to illustrate the benefits of the new estimators and support the simulation results.

**Findings:**

The new estimators outperform the existing estimators in most of the considered scenarios including high and severe cases of multicollinearity. 95% mean prediction interval of all the estimators is also computed for the Tobacco data. The new estimators give the best mean prediction interval among all other estimators.

**The implications of the findings:**

We recommend the use of new estimators to practitioners when the problem of high to severe multicollinearity exists among the predictor variables.

## 1. Introduction

Ordinary least squared (OLS) method of estimation is commonly used in linear regression models. When the problem of multicollinearity exists among the predictor variables then the results obtained by the method of OLS can be misleading [1]. Ridge regression (RR) and Liu regression (LR) suggested by [2, 3] respectively are the two commonly used methods in order to mitigate this problem. LR is usually preferred over RR, because it is the linear function of its shrinkage parameter *d* [4].

The optimal value of shrinkage parameter *d* in LR plays an important role in minimizing the variance. Many researchers have suggested several LR estimators for estimating *d*. Few of them are [4–6]and very recently [7–9]. The existing estimators perform better only when the problem of multicollinearity is not very high. In case of very high or severe multicollinearity, the existing estimators do not perform well in terms of smaller mean squared error (MSE) and mean absolute error (MAE) respectively. To overcome this problem, it was necessary to develop some new estimators.

Therefore, the objective of this paper is to propose some new estimators that are robust to the presence of very high to severe level of multicollinearity. In this paper, the performance of some existing LR estimators is investigated and some new estimators for shrinkage parameter *d* are proposed. The new proposed estimators give the optimal choice of shrinkage parameter and are robust to the presence of very high and severe multicollinearity. Also, the new estimators are compared with the existing ones through a Monte Carlo simulation based on MSE and MAE performance criterions. The MSE and MAE of the new estimators is smaller than OLS and other existing LR estimators and outperform in most of the considered scenarios.

Rest of the article is organized distributed as follows. The statistical methodology that includes the model estimation, new proposed and existing LR estimators are discussed in Section 2. The simulation design and results are discussed in Section 3. Section 4 includes the empirical application to demonstrate the benefits of the new estimators. The conclusion of the paper is given in Section 5.

## 2. Statistical methodology

Consider the following multiple linear regression model in matrix form as:

y=Xβ+ε, (1)

where *y* is the vector of response variable with order (*n* × 1), *X* is the fixed design matrix of predictor variables of order (*n* × *p*) and *β* is the *p* × 1 vector of population regression coefficients. *ε* is the vector of random errors with order (*n* × 1). *ε* is distributed as normal with mean *E*(*ε*) = 0 and variance covariance matrix $E\left(\varepsilon \varepsilon^{'}\right)=\sigma^{2}{\text{I}}_{n}$, I_*n*_ is an (*n* × *n*) identity matrix. The OLS estimator of *β* is given below:

$${\hat{\beta }}_{OLS}={\left(X^{\prime }X\right)}^{-1}X^{\prime }Y.$$ (2)

The OLS estimator gives the unbiased and efficient results provided the basic assumptions of the classical linear regression models are satisfied [1]. However, in the presence of multicollinearity, OLS estimator become inefficient and provide large variance [4]. To circumvent such situation, numerous biased estimation methods are available that provide smaller MSE than OLS and LR is one of them. The LR estimator defined in [2] is given below:

$${\hat{\beta }}_{LIU}={\left(X^{\prime }X+I\right)}^{-1}\left(X^{\prime }X+dI\right){\hat{\beta }}_{OLS},\;0\leq d\leq 1,$$ (3)

In the presence of multicollinearity, ${\hat{\beta }}_{LIU}$ provide the smaller MSE than OLS [10]. The optimal choice of shrinkage parameter *d* plays a vital role in minimizing the MSE of ${\hat{\beta }}_{LIU}$. Some existing LR estimators for the shrinkage parameter *d* are given in the following sub-section.

### 2.1 Some existing LR estimators

Consider the canonical form of model (1) as:

y=Zα+ε, (4)

where *Z* = *XD* and $\alpha =(\alpha_{1},\alpha_{2},\ldots ,\alpha_{p}{)}^{\prime }=D^{\prime }\beta ,$ *D* is an orthogonal matrix such that $D^{\prime }D=I$ and $Z^{\prime }Z=D^{\prime }X^{\prime }XD=\Lambda$, $\Lambda =diag(\lambda_{1},\lambda_{2},\ldots ,\lambda_{p})$ consists of the eigen values of the $X^{\prime }X$ matrix. Note here that $MSE(\hat{\alpha })=MSE(\hat{\beta })$ so it suffices to consider the canonical form only. The OLS estimator can be defined in canonical form as follows:

$${\hat{\alpha }}_{OLS}=\Lambda^{-1}Z^{\prime }y,$$ (5)

The LR estimator is defined as:

$${\hat{\alpha }}_{LIU}={\left(\Lambda +I\right)}^{-1}(\Lambda +dI){\hat{\alpha }}_{OLS},$$ (6)

The first estimator for *d* was suggested by [2] and is given below:

$${\hat{d}}_{j}=\frac{{\hat{\alpha }}_{j}^{2}-{\hat{\sigma }}^{2}}{\frac{{\hat{\sigma }}^{2}}{\lambda_{j}}+{\hat{\alpha }}_{j}^{2}}$$ (7)

where ${\hat{\alpha }}_{j}$ is the j^th^ element of $\hat{\alpha }$, an OLS estimator of *α*. ${\hat{\sigma }}^{2}$ is the unbiased estimator of population error variance *σ*^2^ and *λ*_*j*_ is the j^th^ eigen value of the matrix $X^{\prime }X$. Liu in [2] also suggested the following estimator:

$$D1=\max(0,\frac{{\hat{\alpha }}_{\max}^{2}-{\hat{\sigma }}^{2}}{\frac{1}{\lambda_{\max}}+{\hat{\alpha }}_{\max}^{2}})$$ (8)

Shukur et al., [6] considered the idea of [5, 11] and suggested the following three estimators:

$$D2=\max(0,median(\frac{{\hat{\alpha }}_{j}^{2}-{\hat{\sigma }}^{2}}{\frac{1}{\lambda_{j}}+{\hat{\alpha }}_{j}^{2}}))$$ (9)

$$D3=\max(0,\frac{1}{p}\sum_{j=1}^{p}\left(\frac{{\hat{\alpha }}_{j}^{2}-{\hat{\sigma }}^{2}}{\frac{1}{\lambda_{j}}+{\hat{\alpha }}_{j}^{2}}\right))$$ (10)

$$D4=\max(0,\max(\frac{{\hat{\alpha }}_{j}^{2}-{\hat{\sigma }}^{2}}{\frac{1}{\lambda_{j}}+{\hat{\alpha }}_{j}^{2}}))$$ (11)

Shukur et al., [6] also suggested the following estimators:

$${\hat{q}}_{j}=\frac{{\hat{\alpha }}_{j}^{2}-1}{\max(\frac{1}{\lambda_{j}})+{\hat{\alpha }}_{j}^{2}}$$ (12)

$$D5=\max(0,median({\hat{q}}_{j}))$$ (13)

$$D6=\max(0,\frac{1}{p}\sum_{j=1}^{p}\left({\hat{q}}_{j}\right))$$ (14)

$$D7=\max(0,\max({\hat{q}}_{j}))$$ (15)

Based on the work of [4, 7], we proposed three new LR estimators in the section to follow.

### 2.2 Proposed method

Following the idea of [4, 7], we propose the following new estimator:

$${\hat{d}}_{\gamma }=\frac{{\left({\hat{\alpha }}_{j}\right)}_{\gamma }-{\hat{\sigma }}^{2}}{\max(\frac{{\hat{\sigma }}^{2}}{\lambda_{j}})+\max({\hat{\alpha }}_{j}^{2})}$$ (16)

where *‘γ’* is the quantile probability. In order to obtain the minimum MSE and MAE, the new estimator ${\hat{d}}_{\gamma }$ depends on the quantile probability whose value is selected according to the level of multicollinearity [7]. Since the range of shrinkage parameter must be between zero and one, therefore we rewrite the proposed estimator as:

$$D\gamma =\max(0,{\hat{d}}_{\gamma })=\max(0,\frac{{\left({\hat{\alpha }}_{j}\right)}_{\gamma }-{\hat{\sigma }}^{2}}{\max(\frac{{\hat{\sigma }}^{2}}{\lambda_{j}})+\max({\hat{\alpha }}_{j}^{2})})$$ (17)

Eq (17) satisfies the interval condition for shrinkage parameter *d* suggested by [2]. In order to present the role of quantile probability, we choose some specific values for ‘*γ*’ as: *0 (minimum)*, *0*.*25 (first quartile)* and *0*.*50 (median)*. The mathematical form of three new LR estimators obtained is given below:

$$D8=\max(0,{\hat{d}}_{0.0})=\max(0,\frac{{\left({\hat{\alpha }}_{j}\right)}_{0.0}-{\hat{\sigma }}^{2}}{\max(\frac{{\hat{\sigma }}^{2}}{\lambda_{j}})+\max({\hat{\alpha }}_{j}^{2})})$$ (18)

$$D9=\max(0,{\hat{d}}_{0.25})=\max(0,\frac{{\left({\hat{\alpha }}_{j}\right)}_{0.25}-{\hat{\sigma }}^{2}}{\max(\frac{{\hat{\sigma }}^{2}}{\lambda_{j}})+\max({\hat{\alpha }}_{j}^{2})})$$ (19)

$$D10=\max(0,{\hat{d}}_{0.50})=\max(0,\frac{{\left({\hat{\alpha }}_{j}\right)}_{0.50}-{\hat{\sigma }}^{2}}{\max(\frac{{\hat{\sigma }}^{2}}{\lambda_{j}})+\max({\hat{\alpha }}_{j}^{2})})$$ (20)

The procedure for generating and analyzing data is given in the next section.

## 3. The design of an experiment

In this section Monte Carlo simulation experiment, a commonly used procedure in literature for the data generation and analysis, Performance evaluation criterion and results are also discussed in this section.

### 3.1 The Monte Carlo simulation

In this section, the performance of LR estimators is compared through extensive simulations. Following [12], The predictor variables are generated as:

$$x_{ij}={\left(1-\rho^{2}\right)}^{1/2}z_{ij}+\rho z_{i(j+1)},\;i=1,2,\ldots ,n,\;j=1,2,\ldots ,p,$$ (21)

where *ρ* is the degree or level of multicollinearity between the predictor variables and are given as *0*.*90*, *0*.*99*, *0*.*999* and *0*.*9999*. *z*_*ij*_ are the random numbers obtained from the standard normal distribution. The *n* observations on the response variable are computed as:

$$y_{i}=\beta_{0}+\beta_{1}x_{i1}+\beta_{2}x_{i2}+\ldots +\beta_{p}x_{ip}+\varepsilon_{i},\;i=1,2,\ldots ,n,$$ (22)

where ε_*i*_ ~*N*(0,*σ*^2^)0, *σ*^2^ is the error variance. *β*_*0*_ is considered to be identically zero. Following [11], the eigen vector corresponding to maximum eigen value of the $X^{\prime }X$ matrix is taken as the vector of regression coefficients. Following [4–6] the different factors we choose to vary in our study are given below:

Error variance: *σ*^2^ = 0.5, 1, 2
Sample size: *n* = 25, 50, 100, 200
Predictor variables: *p* = 4, 8, 16, 32
Multicollinearity: *ρ* = 0.90, 0.99, 0.999, 0.9999

### 3.2 Performance evaluation criteria

**Fig 1:**
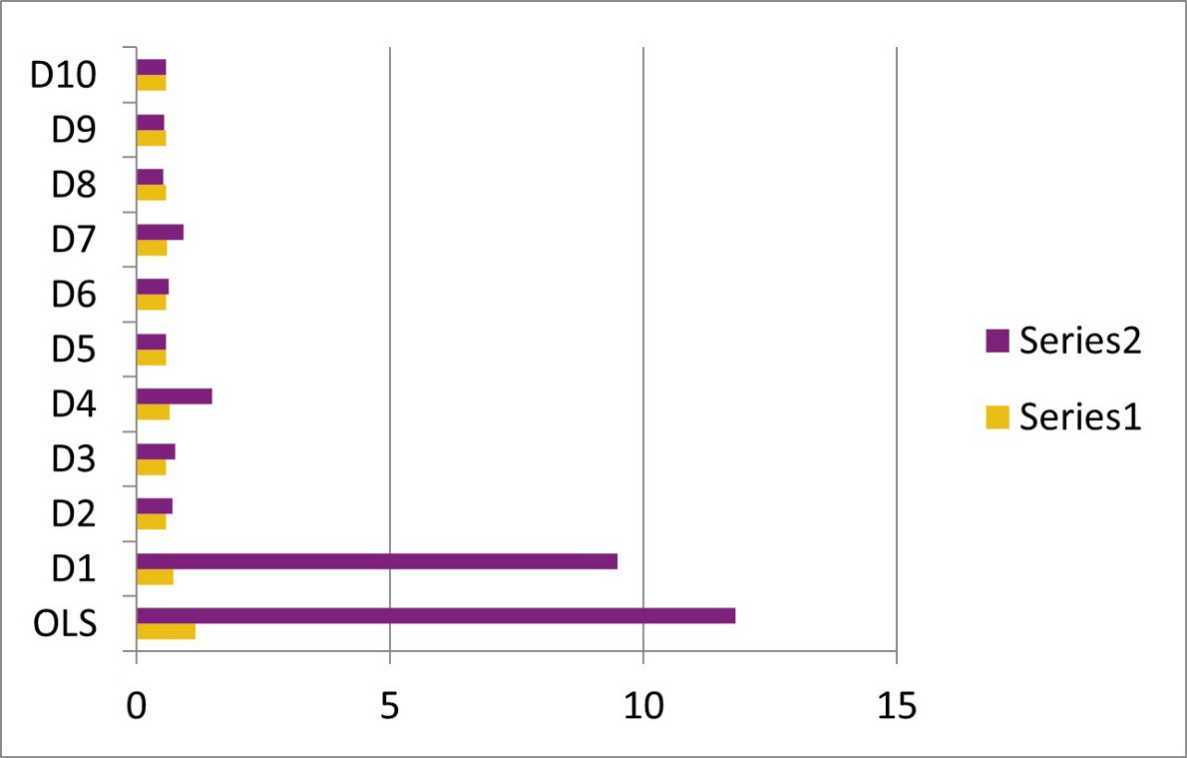
EMSE values for n = 25, p = 4 and ~*N*(0,1).

Following [4], MSE and MAE criterions are used to judge the performance of the different LR estimators. Estimated MSE (EMSE) and MAE (EMAE) are defined as:

$$\text{MSE}=\frac{\sum_{\text{i}=1}^{\text{M}}{\left({\hat{\beta }}_{\text{i}}-\beta \right)}^{\text{'}}\left({\hat{\beta }}_{\text{i}}-\beta \right)}{\text{M}},$$ (23)

$$\text{MAE}=\frac{\sum_{\text{i}=1}^{\text{M}}\left|{\hat{\beta }}_{\text{i}}-\beta \right|}{\text{M}},$$ (24)

where $\hat{\beta_{i}}$ is the estimated value of *β*. M shows the simulation runs. In this study we choose M = 5000. The EMSE simulation results are presented in Tables 1–3 and Fig 1 and EMAE in Tables 4–6. The results are discussed in the section to follow.

**Table 1:** EMSE with *ϵ*~*N*(0,1) and *p = 4*.

*ρ*	0.90	0.99	0.999	0.9999	0.90	0.99	0.999	0.9999
*n*	25				50			
OLS	1.1596	11.8198	119.5606	1185.9839	0.8299	8.8919	87.0138	860.7005
D1	0.7348	9.4928	116.0692	1182.3169	0.5410	6.9544	83.7579	857.1885
D2	0.5855	0.7127	0.8423	5.3231	0.4518	0.6859	0.4039	1.2142
D3	0.5895	0.7627	1.0614	7.7797	0.4531	0.7210	0.5467	2.2779
D4	0.6466	1.4899	5.9986	55.1485	0.4803	1.0350	2.4828	18.0555
D5	0.5816	0.5747	0.2235	0.5026	0.4515	0.6364	0.1841	0.0782
D6	0.5825	0.6304	0.3973	1.5679	0.4518	0.6599	0.2436	0.3090
D7	0.6030	0.9241	2.0217	14.7750	0.4592	0.7610	0.6822	3.0010
D8	0.5812	0.5340	0.1000	0.0200	0.4514	0.6289	0.1564	0.0222
D9	0.5814	0.5434	0.1310	0.1171	0.4514	0.6302	0.1637	0.0351
D10	0.5827	0.5783	0.2271	0.5706	0.4515	0.6368	0.1852	0.0800
*n*	100				200			
OLS	0.3009	3.1852	32.7389	316.5423	0.1701	1.7969	17.8274	178.7094
D1	0.2380	2.0586	29.8277	313.0200	0.1454	1.0979	15.3159	175.2989
D2	0.2332	0.6859	0.3835	0.2193	0.1443	0.5792	0.4920	0.1406
D3	0.2332	0.6988	0.4384	0.4404	0.1443	0.5849	0.5199	0.2306
D4	0.2367	0.7789	0.9362	3.2281	0.1453	0.6167	0.7018	1.1503
D5	0.2332	0.6789	0.3261	0.0616	0.1443	0.5781	0.4719	0.0886
D6	0.2332	0.6837	0.3453	0.0989	0.1443	0.5792	0.4795	0.1022
D7	0.2335	0.7062	0.4353	0.4108	0.1443	0.5855	0.5092	0.1854
D8	0.2332	0.6779	0.3145	0.0425	0.1443	0.5780	0.4685	0.0816
D9	0.2332	0.6780	0.3176	0.0474	0.1443	0.5780	0.4693	0.0835
D10	0.2332	0.6790	0.3262	0.0616	0.1443	0.5781	0.4720	0.0886

**Table 2:** EMSE with *ϵ*~*N*(0,1) and *p = 8*.

*ρ*	0.90	0.99	0.999	0.9999	0.90	0.99	0.999	0.9999
*n*	25				50			
OLS	3.8894	41.4047	417.5922	4181.4422	1.4009	14.2396	140.7281	1416.1652
D1	2.7308	38.0793	413.4239	4177.1657	0.9652	11.6778	136.5833	1411.7423
D2	1.1823	1.3724	1.1818	6.3546	0.7914	1.5760	0.7326	0.7972
D3	1.1869	1.5626	2.1583	14.6674	0.7915	1.6281	0.9376	1.9487
D4	1.5029	4.5126	25.7986	243.7856	0.8400	2.2729	4.9325	35.3770
D5	1.1814	1.2202	0.2996	0.1336	0.7914	1.5565	0.5517	0.0989
D6	1.1860	1.2945	0.5072	1.3767	0.7915	1.5763	0.6083	0.2559
D7	1.2983	2.0319	5.3209	47.7690	0.8029	1.7414	1.2556	4.5674
D8	1.1814	1.2077	0.2559	0.0331	0.7914	1.5546	0.5315	0.0663
D9	1.1814	1.2096	0.2654	0.0500	0.7914	1.5547	0.5354	0.0735
D10	1.1816	1.2225	0.3035	0.1465	0.7914	1.5570	0.5522	0.1002
*n*	100				200			
OLS	0.7568	7.8630	76.8840	778.2603	0.2985	3.1436	31.8130	319.6636
D1	0.5552	6.0314	73.0770	773.9426	0.2538	2.1488	28.5423	315.3435
D2	0.5110	1.4841	0.9194	0.2891	0.2514	1.1341	1.3510	0.3020
D3	0.5110	1.4998	1.0103	0.6101	0.2514	1.1362	1.3915	0.4036
D4	0.5239	1.7211	2.3074	9.3779	0.2532	1.1944	1.8143	2.5146
D5	0.5110	1.4826	0.8593	0.1329	0.2514	1.1341	1.3357	0.2545
D6	0.5110	1.4877	0.8814	0.1743	0.2514	1.1346	1.3438	0.2693
D7	0.5131	1.5394	1.0682	1.0295	0.2514	1.1454	1.4004	0.4309
D8	0.5110	1.4825	0.8535	0.1223	0.2514	1.1341	1.3340	0.2494
D9	0.5110	1.4825	0.8544	0.1248	0.2514	1.1341	1.3342	0.2506
D10	0.5110	1.4826	0.8594	0.1330	0.2514	1.1341	1.3357	0.2546

**Table 3:** EMSE with *n = 100* and *ϵ* ~ *N*(0, σ).

*σ*	0.5				2			
*ρ*	0.90	0.99	0.999	0.9999	0.90	0.99	0.999	0.9999
*p*	16							
OLS	0.4392	4.7232	46.7018	462.2520	6.9753	75.0982	742.4007	7373.3433
D1	0.3763	4.0681	45.2599	460.6382	5.0758	64.6146	723.5484	7352.3531
D2	0.2552	0.8122	0.4619	0.0769	4.0668	13.0077	8.4506	6.6499
D3	0.2562	0.8783	0.7998	1.3977	4.0668	13.0061	7.1471	1.0051
D4	0.3736	3.0591	24.6822	232.2378	4.1879	16.5939	39.7142	241.8414
D5	0.2552	0.8122	0.4548	0.0637	4.0668	13.0163	7.2507	1.1671
D6	0.2552	0.8122	0.4561	0.0657	4.0687	13.1268	7.6249	2.0437
D7	0.2553	0.8173	0.4743	0.1162	4.2435	14.9294	17.7729	77.2964
D8	0.2552	0.8122	0.4547	0.0632	4.0668	13.0061	7.1471	1.0048
D9	0.2552	0.8122	0.4550	0.0638	4.0668	13.0061	7.1487	1.0129
D10	0.2552	0.8122	0.4563	0.0656	4.0668	13.0062	7.1666	1.0424
*p*	32							
OLS	1.2476	13.3404	137.5117	1351.8114	19.8422	210.8066	2130.6648	21882.2836
D1	1.0580	12.4017	135.9954	1350.1507	15.4628	196.4901	2108.5013	21858.3221
D2	0.5341	1.6520	0.9365	0.1568	8.5682	26.4054	17.3436	14.9942
D3	0.5341	1.7210	1.2869	1.3676	8.5682	26.4050	14.7543	2.1536
D4	0.9801	8.4658	75.5821	720.4845	9.0544	37.2481	122.1283	894.3546
D5	0.5341	1.6520	0.9253	0.1339	8.5682	26.4103	14.8186	2.2314
D6	0.5341	1.6521	0.9266	0.1358	8.5732	26.5112	15.1745	3.0021
D7	0.5353	1.6621	0.9665	0.2504	9.0945	30.3438	41.8560	239.1411
D8	0.5341	1.6520	0.9253	0.1336	8.5682	26.4050	14.7514	2.1318
D9	0.5341	1.6520	0.9253	0.1339	8.5682	26.4050	14.7520	2.1366
D10	0.5341	1.6520	0.9262	0.1351	8.5682	26.4050	14.7641	2.1559

**Table 4:** EMAE with *ϵ*~*N*(0,1) and *p = 4*.

*ρ*	0.90	0.99	0.999	0.9999	0.90	0.99	0.999	0.9999
*n*	25				50			
OLS	1.7028	5.3848	17.1241	53.8792	1.4083	4.5591	14.1983	44.9883
D1	1.2204	3.9504	14.3493	45.9810	1.0159	3.1746	11.5845	37.6036
D2	1.1298	1.1950	1.0779	2.1735	0.9656	1.1714	0.8180	1.0717
D3	1.1335	1.2403	1.1869	2.5672	0.9667	1.2038	0.9260	1.4128
D4	1.1783	1.6452	2.7749	7.7409	0.9889	1.4055	1.7821	4.2252
D5	1.1277	1.1049	0.6551	0.7595	0.9655	1.1371	0.6125	0.3721
D6	1.1281	1.1428	0.8046	1.2361	0.9656	1.1536	0.6809	0.5866
D7	1.1396	1.3017	1.4257	3.2133	0.9694	1.2159	0.9296	1.3739
D8	1.1275	1.0744	0.4854	0.2126	0.9654	1.1315	0.5704	0.2287
D9	1.1276	1.0817	0.5370	0.3910	0.9654	1.1325	0.5823	0.2736
D10	1.1284	1.1081	0.6587	0.7841	0.9655	1.1375	0.6137	0.3748
*n*	100				200			
OLS	0.8559	2.7624	8.8210	27.4912	0.6342	2.0584	6.4741	20.3757
D1	0.6913	1.7676	6.9137	23.0382	0.5257	1.3097	4.7890	16.8624
D2	0.6865	1.1808	0.8602	0.5641	0.5242	1.0770	0.9718	0.5020
D3	0.6865	1.1920	0.9181	0.7421	0.5242	1.0817	1.0030	0.6084
D4	0.6907	1.2497	1.2543	1.8616	0.5256	1.1055	1.1494	1.1585
D5	0.6865	1.1767	0.8035	0.3557	0.5242	1.0765	0.9539	0.4183
D6	0.6865	1.1792	0.8242	0.4217	0.5242	1.0770	0.9610	0.4447
D7	0.6867	1.1918	0.9005	0.6703	0.5242	1.0803	0.9864	0.5398
D8	0.6865	1.1761	0.7907	0.3054	0.5242	1.0765	0.9508	0.4025
D9	0.6865	1.1761	0.7941	0.3201	0.5242	1.0765	0.9515	0.4072
D10	0.6865	1.1767	0.8037	0.3557	0.5242	1.0765	0.9540	0.4183

**Table 5:** EMAE with *ϵ*~*N*(0,1) and *p = 8*.

*ρ*	0.90	0.99	0.999	0.9999	0.90	0.99	0.999	0.9999
*n*	25				50			
OLS	4.1296	13.3391	42.3151	134.1307	2.5110	7.8695	24.8766	78.4490
D1	3.0313	11.1518	37.4148	118.9553	1.9623	6.2652	22.1038	71.1415
D2	2.3261	2.4613	2.0456	3.7574	1.8611	2.6684	1.7737	1.5440
D3	2.3295	2.6201	2.5752	5.3934	1.8611	2.7123	1.9722	2.1652
D4	2.5410	4.1018	8.2038	23.7107	1.9016	3.1461	3.9945	9.0276
D5	2.3254	2.3231	1.1459	0.7185	1.8611	2.6536	1.5450	0.6682
D6	2.3277	2.3974	1.4279	1.6307	1.8611	2.6688	1.6263	0.9455
D7	2.3893	2.8380	3.0664	6.9472	1.8674	2.7799	2.1030	2.5796
D8	2.3254	2.3101	1.0484	0.3850	1.8611	2.6522	1.5137	0.5489
D9	2.3254	2.3122	1.0712	0.4712	1.8611	2.6523	1.5197	0.5795
D10	2.3256	2.3257	1.1528	0.7369	1.8611	2.6541	1.5458	0.6709
*n*	100				200			
OLS	1.8177	5.7500	17.9344	57.0037	1.1515	3.6650	11.5925	36.6934
D1	1.4863	4.3472	15.6157	51.2633	1.0127	2.7203	9.7871	33.1336
D2	1.4590	2.5875	1.9964	1.0790	1.0100	2.2560	2.4212	1.1473
D3	1.4590	2.6006	2.0950	1.4038	1.0100	2.2577	2.4616	1.2956
D4	1.4715	2.7657	2.9841	4.5945	1.0124	2.3046	2.7971	2.6540
D5	1.4590	2.5864	1.9260	0.7665	1.0100	2.2560	2.4070	1.0523
D6	1.4590	2.5897	1.9539	0.8643	1.0100	2.2562	2.4149	1.0839
D7	1.4601	2.6237	2.1233	1.4636	1.0101	2.2624	2.4646	1.2779
D8	1.4590	2.5863	1.9189	0.7323	1.0100	2.2560	2.4055	1.0407
D9	1.4590	2.5863	1.9200	0.7406	1.0100	2.2560	2.4057	1.0434
D10	1.4590	2.5864	1.9261	0.7668	1.0100	2.2560	2.4070	1.0524

**Table 6:** EMAE with *n = 100* and *ϵ*~*N*(0,*σ*).

***σ***	0.5				2			
*ρ*	0.90	0.99	0.999	0.9999	0.90	0.99	0.999	0.9999
*p*	16							
OLS	1.8681	5.9671	18.9033	59.2703	7.4724	23.8839	75.1942	236.5916
D1	1.6846	5.1351	17.3340	55.4982	6.2488	20.2901	69.1871	221.6485
D2	1.5014	2.7710	2.0561	0.8446	6.0071	11.1240	8.8420	7.0330
D3	1.5032	2.8909	2.7621	3.3132	6.0071	11.1235	8.0795	3.0193
D4	1.6832	4.7629	13.1063	39.4865	6.0381	12.3402	17.3253	36.6493
D5	1.5014	2.7710	2.0374	0.7611	6.0071	11.1281	8.1490	3.2867
D6	1.5014	2.7710	2.0411	0.7754	6.0075	11.1754	8.3905	4.1168
D7	1.5015	2.7792	2.0875	0.9369	6.0590	11.7235	11.0411	13.2700
D8	1.5014	2.7710	2.0372	0.7575	6.0071	11.1235	8.0795	3.0190
D9	1.5014	2.7710	2.0379	0.7613	6.0071	11.1235	8.0806	3.0336
D10	1.5014	2.7710	2.0415	0.7741	6.0071	11.1235	8.0925	3.0853
*p*	32							
OLS	4.1416	13.0857	41.6043	131.3138	16.5865	52.2138	165.5365	526.9623
D1	3.6986	11.6403	38.4959	122.3703	14.0024	46.4712	153.0526	490.7571
D2	3.1291	5.6694	4.1787	1.7127	12.5223	22.6677	18.1595	14.9002
D3	3.1292	5.8057	5.0067	4.5977	12.5223	22.6675	16.5426	6.2201
D4	3.6652	10.4266	29.3906	89.4904	12.6656	26.2932	41.2662	95.4248
D5	3.1291	5.6694	4.1459	1.5543	12.5223	22.6701	16.5903	6.3920
D6	3.1291	5.6695	4.1502	1.5697	12.5236	22.7221	16.8505	7.3205
D7	3.1304	5.6894	4.2554	1.9168	12.6807	23.8919	22.5386	27.7401
D8	3.1291	5.6694	4.1458	1.5517	12.5223	22.6675	16.5410	6.2033
D9	3.1291	5.6694	4.1460	1.5542	12.5223	22.6675	16.5414	6.2127
D10	3.1291	5.6694	4.1486	1.5634	12.5223	22.6675	16.5502	6.2501

### 3.3 Results and discussion

The EMSE and EMAE values of the new and existing LR estimators are presented in Tables 1–6 and Fig 1 respectively. The performance of the LR estimators is evaluated with respect to different factors such as multicollinearity, error variance, sample size and the predictor variables. These factors affect the simulation design [8]. The effect of each factor on EMSE and EMAE of estimators are discussed below:

Multicollinearity: Increase in the level of multicollinearity increases the EMSE and EMAE of all the estimators. The performance of OLS estimator deteriorates when the multicollinearity becomes very high. LR estimators outperform OLS for all the levels of multicollinearity. However, among all LR estimators, the EMSE and EMAE of the proposed estimators D8-D10 is generally smaller than existing estimators. While the estimator D5 remain close competitor to the proposed estimators only in the case mild to high multicollinearity. But in case of high to severe multicollinearity only the proposed estimators outperform. Fig 1 also support the proposed estimators.

**Sample size:** Increase in the sample size generally decreases the EMSE and EMAE of all the estimators. But the variation in the sample size does not alter the best performance of proposed estimators as observed in the case of multicollinearity.

**Predictors:** When the number of predictors increases the EMSE and EMAE of all the estimator’s increases. But the performance pattern of the estimators remains same as in the case of multicollinearity and sample size. It is also seen from the tables that increase in the EMSE of OLS estimator is relatively higher than all LR estimators. LR with Liu parameter D8 exhibits the lowest EMSE and EMAE.

Error variance or Standard deviation: The EMSE and EMAE of the estimators increases with the increase in the value of error variance. However, the performance of proposed estimators is better than other existing estimators.

The concluded remarks from Tables 1–6 are that the new LR estimators D8-D10 perform efficiently than the other existing LR estimators particularly in the case high to severe multicollinearity. The new estimators also outperform the OLS estimator substantially. Therefore, it is concluded that the new estimators D8-D10 outperform in terms of smaller EMSE and EMAE. Also, among new estimators, the new estimator D8 is more efficient and is the best choice for the practitioners in the presence of high and severe multicollinearity.

## 4. Applications

In the previous section, the performance of estimators is evaluated through Monte Carlo simulation experiment where some ideal conditions are assumed. Contrary to the simulation study, in this section, a numerical example of Tobacco dataset taken from [13] is considered to evaluate the estimators in real world problems.

### 4.1 Tobacco data

The first numerical example used in this study is the Tobacco dataset taken from [13] to compare the performance of new estimators in applied scenario. This data has already been used in literature, see e.g., [14]. The dataset consists of 30 observations of tobacco blends. The percentage concentrations of four important components are considered as predictor variables and the amount of heat given off by the tobacco during the smoking process as a response variable. The model for this dataset is defined as:

$$y=\beta_{0}+\beta_{1}X_{1}+\beta_{2}X_{2}+\beta_{3}X_{3}+\beta_{4}X_{4}+\varepsilon$$ (25)

Condition number (CN) is used to measure the severity of multicollinearity among predictor variables [15] given as:

CN=λmaxλmin,

where *λ*_max_ and *λ*_min_ are the maximum and minimum eigen values of the matrix $X^{\prime }X$ respectively. Following [1], a rule of thumb is that multicollinearity is moderate if the CN is between 10 and 30, high if it is between 30 and 100 and severe when it is greater than 100. The CN for this dataset is 43.50096, which shows that high multicollinearity exists among the predictor variables. The Shapiro-Wilk (W) normality test is used to test the normality of response variable. We obtain the value test statistic W = 0.91248 and P-value = 0.06719 which shows that the response variable is normal at 5% level of significance. MSE of OLS and Liu estimators from [2] can be written as:

$$MSE(\hat{\alpha })=\sigma^{2}\sum_{j=1}^{p}\frac{1}{\lambda_{j}},$$

$$MSE(\hat{\alpha }(d))=\sigma^{2}\sum_{j=1}^{p}\frac{{\left(\lambda_{j}+d\right)}^{2}}{\lambda_{j}{\left(\lambda_{j}+1\right)}^{2}}+{\left(d-1\right)}^{2}\sum_{j=1}^{p}\frac{{\hat{\alpha }}_{j}^{2}}{{\left(\lambda_{j}+1\right)}^{2}},$$

The estimated values for *d*, regression coefficients and MSE of estimators are presented in Table 7. This table shows that the LR estimators have smaller MSE than OLS. However, among the LR estimators, new estimator D8 outperform and therefore highly efficient among others.

**Table 7:** Estimated values for *d*, MSE and regression coefficients of Tobacco data.

Estimators	*d*	MSE	${\hat{\beta }}_{1}$	${\hat{\beta }}_{2}$	${\hat{\beta }}_{3}$	${\hat{\beta }}_{4}$
OLS	--	32.4972	0.4857	-0.6728	1.0744	1.4438
D1	0.8719	24.7963	0.4852	-0.6157	0.9582	1.2696
D2	0.0070	3.6233	0.4815	-0.2303	0.1742	0.0940
D3	0.1011	3.3260	0.4819	-0.2722	0.2594	0.2219
D4	0.3859	6.2963	0.4831	-0.3992	0.5177	0.6091
D5	0.0000	3.6710	0.4815	-0.2272	0.1678	0.0844
D6	0.0000	3.6710	0.4815	-0.2272	0.1678	0.0844
D7	0.0023	3.6549	0.4815	-0.2282	0.1699	0.0876
D8	0.0077	3.3188	0.4815	-0.2306	0.1748	0.0949
D9	0.0144	3.5772	0.4816	-0.2336	0.1808	0.1040
D10	0.0310	3.4872	0.4816	-0.2410	0.1959	0.1266

### 4.2 Prediction interval

In this section, 95% mean prediction interval of all the estimators is computed from the Tobacco dataset. We consider the following values of predictor variables: X_0_^*’*^ = (X_10_, X_20_, X_30_, X_40_) = (20.6, 10.9, 33.62, 39.76). 100(1-α)% mean prediction interval for the response variable is given as:

$$OLS:\;{\hat{y}}_{0}\pm t_{1-\frac{\alpha }{2},\;v}\sqrt{var\left({\hat{y}}_{0}\right)},$$ (26)

$$LIU:\;{\tilde{y}}_{0}\pm t_{1-\frac{\alpha }{2},\;v}\sqrt{var\left({\tilde{y}}_{0}\right)},$$ (27)

where ${\hat{y}}_{0}={\hat{\beta }}_{1}X_{10}+{\hat{\beta }}_{2}X_{20}+{\hat{\beta }}_{3}X_{30}+{\hat{\beta }}_{4}X_{40}$, ${\tilde{y}}_{0}={\tilde{\beta }}_{1}X_{10}+{\tilde{\beta }}_{2}X_{20}+{\tilde{\beta }}_{3}X_{30}+{\tilde{\beta }}_{4}X_{40}$, $\hat{\beta }$ and $\tilde{\beta }$ are the OLS and Liu estimators respectively. $t_{1-\frac{\alpha }{2}}$ is the $\left(1-\frac{\alpha }{2}\right)$ quantile from the Student’s t-distribution with $\left(v=n-p\right)$ degrees of freedom. $var\left({\hat{y}}_{0}\right)={\hat{\sigma }}^{2}X_{0}^{'}{\left(X^{\prime }X\right)}^{-1}X_{0}$ and $var\left({\tilde{y}}_{0}\right)={\hat{\sigma }}^{2}X_{0}^{'}Q_{d}{\left(X^{\prime }X\right)}^{-1}Q_{d}^{'}X_{0}$, $Q_{d}={\left(X^{\prime }X+I\right)}^{-1}\left(X^{\prime }X+dI\right)$. For detail see [1, 4]. The results for the 95% mean prediction interval is given in the Table 8. From this table, we see that the new estimator D8 gives the best mean prediction interval among all other estimators.

**Table 8:** 95% mean prediction interval for the Tobacco data.

Estimators	Lower	Upper	Difference
OLS	71.6436	120.7570	49.1134
D1	64.1423	107.8168	43.6745
D2	12.2255	21.7781	9.5526
D3	18.2868	30.7197	12.4329
D4	35.5374	58.9073	23.3699
D5	11.7547	21.1265	9.3718
D6	11.7547	21.1265	9.3718
D7	11.9098	21.3403	9.4305
D8	12.4710	21.8415	9.3705
D9	12.7142	22.4621	9.7479
D10	13.8076	24.0194	10.2118

## 5. Concluding remarks

In this paper, some new quantile based LR estimators for the shrinkage parameter ‘*d*’ are proposed in order to minimize the variance and mitigate the problem of multicollinearity. Monte Carlo simulation experiment was performed to compare the performance of estimators. MSE and MAE performance measures were used. Multicollinearity, Sample size, predictor variables and error variance were the different factors we choose to vary in our study. It is concluded that all the LR estimators generally perform better than OLS estimator. Furthermore, among the LR estimators, the new estimators have shown best performance in the simulation and application. The LR is a robust choice than the OLS when the problem of multicollinearity is present. Moreover, among the new estimators, D8 performs better than other considered estimators in many evaluated instances particularly when the problem of multicollinearity is very high and severe. Estimators D5 and D9 were the close competitors to D8. Therefore, we recommend the use of LR method with shrinkage new estimator D8 over OLS when the problem of multicollinearity is present in the data.

### 5.1 Future research

Future research directions: In this research only the problem of multicollinearity is considered. When the outliers are also present in the data then the performance of the estimators will change. In future, we can develop some new robust LR estimators to overcome the joint problem of multicollinearity and outliers.

## Supporting information

S1 File (DOCX)
