# Expression of Concern: Modified Liu estimators in the linear regression model: An application to Tobacco data

**DOI:** 10.1371/journal.pone.0343789

**Published:** 2026-03-02

**Authors:** The PLOS One Editors

The *PLOS One* Editors issue this Expression of Concern to alert readers that this article [1] was identified as one of a series of submissions for which we have concerns about adherence to the journal’s authorship policy. We regret that these issues were not addressed prior to the article’s publication.
