# Supplementary material for: Modified Liu estimators in the linear regression model: An application to Tobacco data
Source: PLoS One. 2021 Nov 22;16(11):e0259991. doi: 10.1371/journal.pone.0259991 (PMC8608338; doi:10.1371/journal.pone.0259991)
Supplement: S1 File — (DOCX) [file pone.0259991.s001.docx]

**Technical Supports**

**R-Codes**

**A.Simulation-Codes:**

**#========Quantile-based estimation of Liu estimator in the Linear regression model========**

**set.seed(1234)**

**sigma1 = c(0.5,1,2,5)**

**ssize=c(25,50,100,200)**

**pred=c(4,8)**

**corr=c(0.90,0.99,0.999,0.9999)**

**N=5000**

**#-----------------------------------------------------------------------------------------------------**

**MSE.r=matrix(0,11,length(corr))**

**MSE.s=array(0,dim = c(11,length(corr),length(sigma1)))**

**MSE.n=array(0,dim = c(11,length(corr),length(sigma1),length(ssize)))**

**MSE.p=array(0,dim = c(11,length(corr),length(sigma1),length(ssize),length(pred)))**

**MAE.r=matrix(0,11,length(corr))**

**MAE.s=array(0,dim = c(11,length(corr),length(sigma1)))**

**MAE.n=array(0,dim = c(11,length(corr),length(sigma1),length(ssize)))**

**MAE.p=array(0,dim = c(11,length(corr),length(sigma1),length(ssize),length(pred)))**

**#-----------------------------------------------------------------------------------------------------**

**# predictors loop starts here**

**for(b in 1:length(pred)){**

**p=pred[b]**

**# Sample size loop**

**I=diag(p)**

**for(a in 1:length(ssize)){**

**n=ssize[a]**

**# Generating standard normal random numbers**

**z=matrix(0,n,p)**

**for(i in 1:p){ #Generating stanndard normal random variable Z**

**z[,i]=rnorm(n,0,1)**

**}**

**x=matrix(0,n,p)**

**det.corr.x=rep(0,length(corr))**

**# error variance loop**

**for(s in 1:length(sigma1)){**

**sigma=sigma1[s]**

**# Correlation loop**

**for(q in 1:length(corr)){**

**r=corr[q]**

**# Generating matrix of independent variables**

**for(i in 1:p){ #Generating X matrix**

**x[,i]=sqrt(1-(r^2))*z[,i]+r*z[,p]**

**}**

**x=scale(x,center = TRUE,scale = TRUE) #Scaling x,,, standardizing x and y**

**det.corr.x[q]=det(cor(x))**

**eigen(cor(x))**

**ev=eigen(cor(x))$values**

**vec=eigen(cor(x))$vectors**

**which.max(ev)**

**beta=vec[,which.max(ev)]**

**C=t(x)%*%(x)**

**D=eigen(C)$vectors**

**lamb=diag(t(D)%*%C%*%D)**

**xstar=x%*%D**

**alpha=t(D)%*%beta**

**#-----------------------------------------------------------------------------------------------------------**

**betahat_ols=matrix(ncol=N, nrow=p)**

**alphahatD1_M = matrix(ncol=N, nrow=p)**

**alphahatD2_M = matrix(ncol=N, nrow=p)**

**alphahatD3_M = matrix(ncol=N, nrow=p)**

**alphahatD4_M = matrix(ncol=N, nrow=p)**

**alphahatD5_M = matrix(ncol=N, nrow=p)**

**alphahatD6_M = matrix(ncol=N, nrow=p)**

**alphahatD7_M = matrix(ncol=N, nrow=p)**

**alphahatD8_M = matrix(ncol=N, nrow=p)**

**alphahatD9_M = matrix(ncol=N, nrow=p)**

**alphahatD10_M = matrix(ncol=N, nrow=p)**

**#alphahatD11_M = matrix(ncol=N, nrow=p)**

**#alphahatD12_M = matrix(ncol=N, nrow=p)**

**#alphahatD13_M = matrix(ncol=N, nrow=p)**

**#---------------------------------------------------------------------------------------------------------------**

**#Simulation loop starts here**

**for(i in 1:N){**

**e=rnorm(n,0,sigma)**

**y=xstar%*%alpha+e**

**betahat=solve(t(x)%*%x)%*%t(x)%*%y**

**yhat=x%*%betahat**

**sigmahat=(sum((y-yhat)^2))/(n-p)**

**alphahat=solve(diag(lamb))%*%t(xstar)%*%y**

**#OLS method**

**betahat_ols[,i]=c(betahat)**

**#Liu estimator 1993**

**D.liu=(max(alphahat^2)-sigmahat)/(max(alphahat^2)+(1/max(ev)))**

**D1=max(0,D.liu)**

**#Shukur, mansson and sjolander 2015a**

**D.sms=((alphahat^2)-sigmahat)/((alphahat^2)+(1/ev))**

**D2=max(0,median(D.sms))**

**D3=max(0,sum(D.sms)/p)**

**D4=max(0,max(D.sms))**

**#Shukur, mansson and sjolander 2015a**

**Qj=(alphahat^2-1)/(max(1/ev)+(alphahat^2))**

**D5=max(0,median(Qj))**

**D6=max(0,sum(Qj)/p)**

**D7=max(0,max(Qj))**

**#--------------------------------------------------------------------------------------**

**#Proposed estimators**

**D8=max(0,(quantile(alphahat^2, probs = 0.00)-sigmahat)/(max(alphahat^2)+(max(sigmahat/ev))))**

**D9=max(0,(quantile(alphahat^2, probs = 0.25)-sigmahat)/(max(alphahat^2)+(max(sigmahat/ev))))**

**D10=max(0,(quantile(alphahat^2, probs = 0.50)-sigmahat)/(max(alphahat^2)+(max(sigmahat/ev))))**

**#Based on D.sms inner part**

**#D8=max(0,(quantile(alphahat^2, probs = 0.00)-1)/(max(alphahat^2)+(max(1/ev))))**

**#D9=max(0,(quantile(alphahat^2, probs = 0.25)-1)/(max(alphahat^2)+(max(1/ev))))**

**#D10=max(0,(quantile(alphahat^2, probs = 0.50)-1)/(max(alphahat^2)+(max(1/ev))))**

**D.vector=c(D1,D2,D3,D4,D5,D6,D7,D8,D9,D10)**

**#--------------------------------------------------------------------------------------**

**#Liu regression estimators**

**alphahatD1=solve(t(xstar)%*%(xstar)+I)%*%(t(xstar)%*%(xstar)+D1*I)%*%alphahat**

**alphahatD1_M[,i]=c(alphahatD1)**

**alphahatD2=solve(t(xstar)%*%(xstar)+I)%*%(t(xstar)%*%(xstar)+D2*I)%*%alphahat**

**alphahatD2_M[,i]=c(alphahatD2)**

**alphahatD3=solve(t(xstar)%*%(xstar)+I)%*%(t(xstar)%*%(xstar)+D3*I)%*%alphahat**

**alphahatD3_M[,i]=c(alphahatD3)**

**alphahatD4=solve(t(xstar)%*%(xstar)+I)%*%(t(xstar)%*%(xstar)+D4*I)%*%alphahat**

**alphahatD4_M[,i]=c(alphahatD4)**

**alphahatD5=solve(t(xstar)%*%(xstar)+I)%*%(t(xstar)%*%(xstar)+D5*I)%*%alphahat**

**alphahatD5_M[,i]=c(alphahatD5)**

**alphahatD6=solve(t(xstar)%*%(xstar)+I)%*%(t(xstar)%*%(xstar)+D6*I)%*%alphahat**

**alphahatD6_M[,i]=c(alphahatD6)**

**alphahatD7=solve(t(xstar)%*%(xstar)+I)%*%(t(xstar)%*%(xstar)+D7*I)%*%alphahat**

**alphahatD7_M[,i]=c(alphahatD7)**

**alphahatD8=solve(t(xstar)%*%(xstar)+I)%*%(t(xstar)%*%(xstar)+D8*I)%*%alphahat**

**alphahatD8_M[,i]=c(alphahatD8)**

**alphahatD9=solve(t(xstar)%*%(xstar)+I)%*%(t(xstar)%*%(xstar)+D9*I)%*%alphahat**

**alphahatD9_M[,i]=c(alphahatD9)**

**alphahatD10=solve(t(xstar)%*%(xstar)+I)%*%(t(xstar)%*%(xstar)+D10*I)%*%alphahat**

**alphahatD10_M[,i]=c(alphahatD10)**

**#alphahatD11=solve(t(xstar)%*%(xstar)+I)%*%(t(xstar)%*%(xstar)+D11*I)%*%alphahat**

**#alphahatD11_M[,i]=c(alphahatD11)**

**#alphahatD12=solve(t(xstar)%*%(xstar)+I)%*%(t(xstar)%*%(xstar)+D12*I)%*%alphahat**

**#alphahatD12_M[,i]=c(alphahatD12)**

**#alphahatD13=solve(t(xstar)%*%(xstar)+I)%*%(t(xstar)%*%(xstar)+D13*I)%*%alphahat**

**#alphahatD13_M[,i]=c(alphahatD13)**

**#------------------------------------------------------------------------------------------------------**

**} #Loop for N=5000 Runs ends**

**#Mean square error estimation**

**MSE_ols=sum((betahat_ols-c(beta))^2)/N**

**MSE1<-sum((alphahatD1_M-c(alpha))^2)/N**

**MSE2<-sum((alphahatD2_M-c(alpha))^2)/N**

**MSE3<-sum((alphahatD3_M-c(alpha))^2)/N**

**MSE4<-sum((alphahatD4_M-c(alpha))^2)/N**

**MSE5<-sum((alphahatD5_M-c(alpha))^2)/N**

**MSE6<-sum((alphahatD6_M-c(alpha))^2)/N**

**MSE7<-sum((alphahatD7_M-c(alpha))^2)/N**

**MSE8<-sum((alphahatD8_M-c(alpha))^2)/N**

**MSE9<-sum((alphahatD9_M-c(alpha))^2)/N**

**MSE10<-sum((alphahatD10_M-c(alpha))^2)/N**

**MSE=c(MSE_ols,MSE1,MSE2,MSE3,MSE4,MSE5,MSE6,MSE7,MSE8,MSE9,MSE10)**

**MSE=round(MSE,5)**

**as.matrix(MSE)**

**MSE.r[,q]=MSE**

**#----------------------------------------------------------------------------------------------------------------------**

**#Mean absolute error estimation**

**MAE_ols=sum(abs(betahat_ols-c(beta)))/N**

**MAE1<-sum(abs(alphahatD1_M-c(alpha)))/N**

**MAE2<-sum(abs(alphahatD2_M-c(alpha)))/N**

**MAE3<-sum(abs(alphahatD3_M-c(alpha)))/N**

**MAE4<-sum(abs(alphahatD4_M-c(alpha)))/N**

**MAE5<-sum(abs(alphahatD5_M-c(alpha)))/N**

**MAE6<-sum(abs(alphahatD6_M-c(alpha)))/N**

**MAE7<-sum(abs(alphahatD7_M-c(alpha)))/N**

**MAE8<-sum(abs(alphahatD8_M-c(alpha)))/N**

**MAE9<-sum(abs(alphahatD9_M-c(alpha)))/N**

**MAE10<-sum(abs(alphahatD10_M-c(alpha)))/N**

**MAE=c(MAE_ols,MAE1,MAE2,MAE3,MAE4,MAE5,MAE6,MAE7,MAE8,MAE9,MAE10)**

**MAE=round(MAE,5)**

**as.matrix(MAE)**

**MAE.r[,q]=MAE**

**} #End of correlation loop**

**MSE.s[,,s]=MSE.r**

**MAE.s[,,s]=MAE.r**

**} #End of error variance loop**

**MSE.n[,,,a]=MSE.s**

**MAE.n[,,,a]=MAE.s**

**} #End of sample size loop**

**MSE.p[,,,,b]=MSE.n**

**MAE.p[,,,,b]=MAE.n**

**} #End of predictors loop**

**col.names=c("0.90","0.99","0.999","0.9999")**

**row.names=c("OLS","D1","D2","D3","D4","D5","D6","D7","D8","D9","D10")**

**matrix.names=c("0.5","1","2","5")**

**matrix.names2=c("25","50","100","200")**

**matrix.names3=c("4","8")**

**dimnames(MSE.p)=list(row.names,col.names,matrix.names,matrix.names2,matrix.names3)**

**dimnames(MAE.p)=list(row.names,col.names,matrix.names,matrix.names2,matrix.names3)**

**write.csv(MSE.p,file = "E:/1.PhD research/2.Research fellows/Mam Irum research/Paper-I/MSE.Qj.csv")**

**write.csv(MAE.p,file = "E:/1.PhD research/2.Research fellows/Mam Irum research/Paper-I/MAE.Qj.csv")**

**#MSE.p**

**#D.vector**

**B.Tobacco Data real Life Application**

#========Quantile-based estimation of Liu estimator in the Linear regression model========

rm(list=ls())

set.seed(1988)

library(MASS)

p<- **4 #No of explanatory variables**

I=diag(p)

setwd("E:\\1.PhD research\\3.Data\\Real data") #To change working directory

data=read.csv("Tobacco.csv",header=TRUE)

x1=data$x1

x2=data$x2

x3=data$x3

x4=data$x4

n<- length(x1)

x<-cbind(x1,x2,x3,x4)

x=scale(x,center = TRUE,scale = TRUE) #Scaling x,,, standardizing x and y

y=data$y

y=(y-mean(y))/sd(y)

#------------------------------------------

evec=eigen(cor(x))

ev=evec$values

e.vec=evec$vectors

beta=e.vec[,which.max(ev)]

c=t(x)%*%x

D=eigen(c)$vectors

xstar=x%*%D

#Z=x%*%D

lam=t(xstar)%*%xstar

lam=round(lam,4)

lamda=diag(lam)

alpha=t(D)%*%beta

beta.hat=solve(t(x)%*%x)%*%t(x)%*%y

y.hat=x%*%beta.hat

sigmahat=sum((y-y.hat)^2)/(n-p)

alphahat=solve(lam)%*%t(xstar)%*%y

#OLS estimator

alphahat.ols=alphahat

alphahat.ols=c(alphahat.ols)

#Liu estimator 1993

D.liu=(max(alphahat^2)-sigmahat)/(max(alphahat^2)+(1/max(ev)))

D1=max(0,D.liu)

#Shukur, mansson and sjolander 2015a

D.sms=((alphahat^2)-sigmahat)/((alphahat^2)+(1/ev))

D2=max(0,median(D.sms))

D3=max(0,sum(D.sms)/p)

#Khalaf and Shukur 2005

D4=max(0,max(D.sms))

#Shukur, mansson and sjolander 2015a

Qj=(alphahat^2-1)/(max(1/ev)+(alphahat^2))

D5=max(0,median(Qj))

D6=max(0,sum(Qj)/p)

D7=max(0,max(Qj))

#--------------------------------------------------------------------------------------

#Proposed estimators

#Based on D.sms inner part

#D6=max(0,abs(quantile(alphahat^2, probs = 0.00)-sigmahat)/(quantile(alphahat^2, probs = 0.00)+(1/quantile(ev, probs = 0.00))))

#D7=max(0,abs(quantile(alphahat^2, probs = 0.25)-sigmahat)/(quantile(alphahat^2, probs = 0.25)+(1/quantile(ev, probs = 0.25))))

#D8=max(0,abs(quantile(alphahat^2, probs = 0.50)-sigmahat)/(quantile(alphahat^2, probs = 0.50)+(1/quantile(ev, probs = 0.50))))

#D9=max(0,abs(quantile(alphahat^2, probs = 0.75)-sigmahat)/(quantile(alphahat^2, probs = 0.75)+(1/quantile(ev, probs = 0.75))))

#D10=max(0,abs(quantile(alphahat^2, probs = 1)-sigmahat)/(quantile(alphahat^2, probs = 1)+(1/quantile(ev, probs = 1))))

#--------------------------------------------------------------------------------------

#Proposed estimators

D8=max(0,(quantile(alphahat^2, probs = 0.00)-sigmahat)/(max(alphahat^2)+(max(sigmahat/ev))))

D9=max(0,(quantile(alphahat^2, probs = 0.25)-sigmahat)/(max(alphahat^2)+(max(sigmahat/ev))))

D10=max(0,(quantile(alphahat^2, probs = 0.50)-sigmahat)/(max(alphahat^2)+(max(sigmahat/ev))))

#--------------------------------------------------------------------------------------

D.vector=c(D1,D2,D3,D4,D5,D6,D7,D8,D9,D10)

as.vector(D.vector)

#Liu regression estimators

alphahatD1=solve(t(xstar)%*%(xstar)+I)%*%(t(xstar)%*%(xstar)+D1*I)%*%alphahat

alphahatD1=c(alphahatD1)

alphahatD2=solve(t(xstar)%*%(xstar)+I)%*%(t(xstar)%*%(xstar)+D2*I)%*%alphahat

alphahatD2=c(alphahatD2)

alphahatD3=solve(t(xstar)%*%(xstar)+I)%*%(t(xstar)%*%(xstar)+D3*I)%*%alphahat

alphahatD3=c(alphahatD3)

alphahatD4=solve(t(xstar)%*%(xstar)+I)%*%(t(xstar)%*%(xstar)+D4*I)%*%alphahat

alphahatD4=c(alphahatD4)

alphahatD5=solve(t(xstar)%*%(xstar)+I)%*%(t(xstar)%*%(xstar)+D5*I)%*%alphahat

alphahatD5=c(alphahatD5)

alphahatD6=solve(t(xstar)%*%(xstar)+I)%*%(t(xstar)%*%(xstar)+D6*I)%*%alphahat

alphahatD6=c(alphahatD6)

alphahatD7=solve(t(xstar)%*%(xstar)+I)%*%(t(xstar)%*%(xstar)+D7*I)%*%alphahat

alphahatD7=c(alphahatD7)

alphahatD8=solve(t(xstar)%*%(xstar)+I)%*%(t(xstar)%*%(xstar)+D8*I)%*%alphahat

alphahatD8=c(alphahatD8)

alphahatD9=solve(t(xstar)%*%(xstar)+I)%*%(t(xstar)%*%(xstar)+D9*I)%*%alphahat

alphahatD9=c(alphahatD9)

alphahatD10=solve(t(xstar)%*%(xstar)+I)%*%(t(xstar)%*%(xstar)+D10*I)%*%alphahat

alphahatD10=c(alphahatD10)

#Computing regression coefficients

comb.coeff=rbind(alphahat.ols,alphahatD1,alphahatD2,alphahatD3,alphahatD4,alphahatD5,alphahatD6,

alphahatD7,alphahatD8,alphahatD9,alphahatD10)

comb.coeff=round(comb.coeff,5)

write.csv(comb.coeff,file = "E:/1.PhD research/2.Research fellows/Mam Irum research/Paper-I/Results/coef.tobacco.csv")

write.csv(D.vector,file = "E:/1.PhD research/2.Research fellows/Mam Irum research/Paper-I/Results/D-vector.csv")

#--------------------------------------------------------------------------------------------------------------

#Prediction interval

x0=c(20.6,10.9,33.62,39.76)

as.vector(x0)

y0.ols=t(x0)%*%alphahat.ols

y0.D1=t(x0)%*%alphahatD1

y0.D2=t(x0)%*%alphahatD2

y0.D3=t(x0)%*%alphahatD3

y0.D4=t(x0)%*%alphahatD4

y0.D5=t(x0)%*%alphahatD5

y0.D6=t(x0)%*%alphahatD6

y0.D7=t(x0)%*%alphahatD7

y0.D8=t(x0)%*%alphahatD8

y0.D9=t(x0)%*%alphahatD9

y0.D10=t(x0)%*%alphahatD10

y0.vector=c(y0.ols,y0.D1,y0.D2,y0.D3,y0.D4,y0.D5,y0.D6,y0.D7,y0.D8,y0.D9,y0.D10)

y0.vector=as.vector(y0.vector)

var.y0=sigmahat*(t(x0)%*%solve(t(x)%*%x)%*%x0)

se.y0.ols=sqrt(var.y0)

QD1=solve(t(x)%*%x+I)%*%(t(x)%*%x+D1*I)

QD2=solve(t(x)%*%x+I)%*%(t(x)%*%x+D2*I)

QD3=solve(t(x)%*%x+I)%*%(t(x)%*%x+D3*I)

QD4=solve(t(x)%*%x+I)%*%(t(x)%*%x+D4*I)

QD5=solve(t(x)%*%x+I)%*%(t(x)%*%x+D5*I)

QD6=solve(t(x)%*%x+I)%*%(t(x)%*%x+D6*I)

QD7=solve(t(x)%*%x+I)%*%(t(x)%*%x+D7*I)

QD8=solve(t(x)%*%x+I)%*%(t(x)%*%x+D8*I)

QD9=solve(t(x)%*%x+I)%*%(t(x)%*%x+D9*I)

QD10=solve(t(x)%*%x+I)%*%(t(x)%*%x+D10*I)

se.y0.D1=sqrt(sigmahat*(t(x0)%*%QD1%*%solve(t(x)%*%x)%*%t(QD1)%*%x0))

se.y0.D2=sqrt(sigmahat*(t(x0)%*%QD2%*%solve(t(x)%*%x)%*%t(QD2)%*%x0))

se.y0.D3=sqrt(sigmahat*(t(x0)%*%QD3%*%solve(t(x)%*%x)%*%t(QD3)%*%x0))

se.y0.D4=sqrt(sigmahat*(t(x0)%*%QD4%*%solve(t(x)%*%x)%*%t(QD4)%*%x0))

se.y0.D5=sqrt(sigmahat*(t(x0)%*%QD5%*%solve(t(x)%*%x)%*%t(QD5)%*%x0))

se.y0.D6=sqrt(sigmahat*(t(x0)%*%QD6%*%solve(t(x)%*%x)%*%t(QD6)%*%x0))

se.y0.D7=sqrt(sigmahat*(t(x0)%*%QD7%*%solve(t(x)%*%x)%*%t(QD7)%*%x0))

se.y0.D8=sqrt(sigmahat*(t(x0)%*%QD8%*%solve(t(x)%*%x)%*%t(QD8)%*%x0))

se.y0.D9=sqrt(sigmahat*(t(x0)%*%QD9%*%solve(t(x)%*%x)%*%t(QD9)%*%x0))

se.y0.D10=sqrt(sigmahat*(t(x0)%*%QD10%*%solve(t(x)%*%x)%*%t(QD10)%*%x0))

se.y0.vector=c(se.y0.ols,se.y0.D1,se.y0.D2,se.y0.D3,se.y0.D4,se.y0.D5,se.y0.D6,se.y0.D7,se.y0.D8,se.y0.D9,se.y0.D10)

se.y0.vector=as.vector(se.y0.vector)

v.df=n-p

t.table=qt(0.975, df=v.df, lower.tail = TRUE, log.p = FALSE)

LCL_y0=rep(0,length(y0.vector))

UCL_y0=rep(0,length(y0.vector))

for (i in 1:length(y0.vector)) {

LCL_y0[i]=y0.vector[i]-(t.table*se.y0.vector[i])

UCL_y0[i]=y0.vector[i]+(t.table*se.y0.vector[i])

}

Pred.int=round(cbind(LCL_y0,UCL_y0),5)

row.names(Pred.int)=c("OLS","D1","D2","D3","D4","D5","D6","D7","D8","D9","D10")

write.csv(Pred.int,file = "E:/1.PhD research/2.Research fellows/Mam Irum research/Paper-I/Results/Pred.int.tob.csv")

#--------------------------------------------------------------------------------------------------------------

**#MSE computation**

MSE.OLS=sigmahat*sum(1/ev)

#MSE of all Liu estimators

a=rep(0,length(D.vector)); b=rep(0,length(D.vector)) #to obtain MSE of ridge estimators

for(i in 1:length(D.vector)){

a[i]=sum(((ev+D.vector[i])^2)/(ev*((ev+1)^2)))

b[i]=((D.vector[i]-1)^2)*sum((alphahat^2)/((ev+1)^2))

}

MSE.Liu=sigmahat*a+b

MSE=c(MSE.OLS,MSE.Liu)

MSE=as.matrix(MSE)

MSE=round(MSE,4)

row.names(MSE)=c("OLS","D1","D2","D3","D4","D5","D6","D7","D8","D9","D10")

colnames(MSE)=c("MSE")

write.csv(MSE,file = "E:/1.PhD research/2.Research fellows/Mam Irum research/Paper-I/Results/MSE.tobacco.csv")

**#OLS**

xnam <- paste0("x", 1:p)

fmla<- as.formula(paste("y ~ ", paste(xnam, collapse= "+")))

model<-lm(fmla)

sigma2<-sum(model$residuals^2)/(n-p-1)

sigmahat.e=round(sqrt(sigma2),4)

#eigen values

ev1=eigen(cor(x))$values

ev1=round(ev1,4)

max.per.ev1=(max(ev1)/4)*100

max.per.ev1=round(max.per.ev1,2)

cor.x=round(cor(x),4)

cor.y=round(cor(x,y),4)

cor.xy=cbind(cor.x,cor.y)

write.csv(cor.xy,file = "E:/1.PhD research/2.Research fellows/Mam Irum research/Paper-I/Results/Corr.tobacco.csv")

cor.xy

round(det(cor(x)),6)

MSE

ev1

max.per.ev1

sigma2

sigmahat.e

CN=max(ev1)/min(ev1) #Computing condition number

CN1=sqrt(CN)

CN

CN1

#Checking normality of errors

res.ols=model$residuals

hist(res.ols)

#lines(density(res.ols),col="red")

ks.test(res.ols,"pnorm",3)

#library(nortest)

shapiro.test(res.ols)

library(boot)

round(k3.linear(res.ols),4)

#Checking outliers

#Influence measures

inflm.model <- influence.measures(model)

which(apply(inflm.model$is.inf, 1, any)) #outliers

summary(inflm.model) #Summary of outlying observations
